# Visual evoked potentials to a diamond pattern can predict accommodation power more accurately than those to a checkerboard pattern

**DOI:** 10.1371/journal.pone.0349254

**Published:** 2026-05-14

**Authors:** Ryota Yamamoto, Shozo Tobimatsu, Kiyoka Miyahara, Takeshi Yoshitomi

**Affiliations:** 1 Department of Ophthalmology, Faculty of Medicine, Saga University Hospital, Saga, Japan; 2 Department of Orthoptics, Faculty of Medicine, Fukuoka International University of Health and Welfare, Fukuoka, Japan; 3 Maeno Eye Clinic, Kitakyushu, Japan; Midwestern University, UNITED STATES OF AMERICA

## Abstract

**Purpose:**

Visual evoked potentials (VEPs) are significantly influenced by the spatial frequency (SF) and angular distribution of visual stimuli. Here, we examined the effects of retinal blur on VEPs with changes in refractive power, to determine whether VEPs can be used as an index of objective accommodation (OA).

**Methods:**

Twenty-one university students with corrected visual acuity of at least 0.0 logMAR participated in the study. Checkerboard (Check) and Check rotated 45° (Diamond) patterns were used as the visual stimuli. Pattern reversal VEPs were recorded from the uncyclopleged dominant eye. Changes in P100 latency and amplitude were assessed by concave-lens loading to obtain OA. Near and far points of accommodation were determined using a visual display terminal near-point meter to obtain subjective accommodation (SA). Linear regression analysis between SA and OA was performed.

**Results:**

P100 amplitudes were affected by the refractive power even when the P100 latency of VEPs was unaffected. VEPs to Diamond more precisely predicted OA than those to Check: SA was 11.70 ± 3.15 D (mean ± SD) whereas the OAs to Diamond and Check were 5.05 ± 1.15 D and 4.74 ± 1.62 D, respectively, with SA being significantly higher (*p* < 0.01). A significant positive correlation was found between SA and OA for Diamond (*r* = 0.64, *p* = 0.003) and between SA and OA for Check (*r* = 0.54, *p* = 0.016).

**Conclusions:**

VEPs to Diamond predicted the refractive power more accurately than those of Check due to a better fit. Visual resolution is better in response to vertical and horizontal stimuli than oblique ones (orientation effect). Because the fundamental SF component of Check, but not Diamond, lies in the 45° and 135° (oblique) orientations, VEPs to Check are more susceptible to retinal blur, probably due to the orientation effect. SA had significantly greater accommodation values than OA due to the different methodologies.

## Introduction

In 1972, Halliday et al. [1] reported that visual evoked potentials (VEPs) to checkerboard (Check) pattern reversal (PR) stimulation were useful for confirmation of optic nerve disease in patients with multiple sclerosis (MS). Since then, PR-VEPs have been acknowledged for their utility during functional assessment of the primary visual cortex (V1) from the optic nerve [2,3]. It is important to note that the latency and amplitude of P100 are significantly affected by the refractory power of the subjects [4–7], the spatial frequency (SF) [2,7,8] of the check size, and the stimulus orientation of the pattern [9–11].

**Fig 1:**
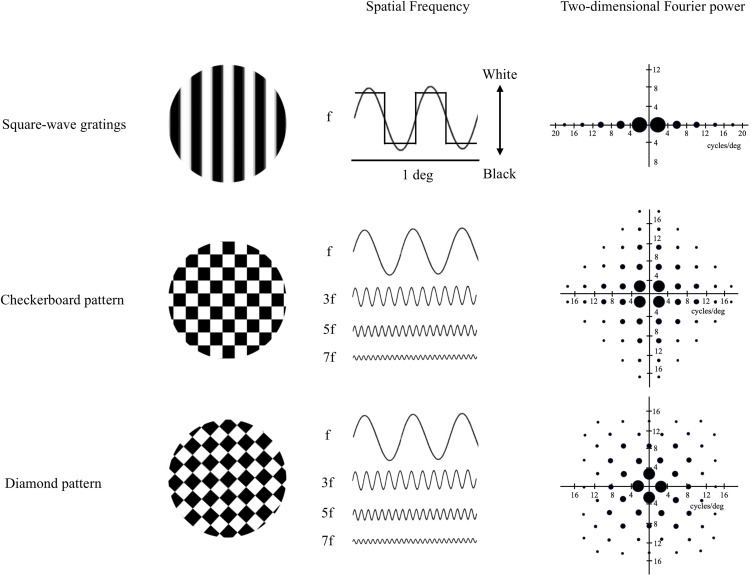
Schematic drawings of the two-dimensional Fourier spectra of the square-wave gratings, Check and Diamond patterns. The magnitudes of the Fourier components are represented by the areas of the filled circles. The Fourier spectrum of the square-wave gratings with 15-min stripes shows that the fundamental spatial frequency (SF) is 2 cycles/deg and its spatial distribution is horizontal with additional odd harmonic components such as the third, fifth and seventh harmonics of the fundamental SF (top). The Fourier spectrum of Check with 15-min squares shows that the fundamental components are located on the diagonal meridians at 2.8 cycles/deg from the origin (√2 times the frequency), whereas other harmonics are widely distributed throughout the SF plane (middle). In the Fourier spectrum of Diamond, the edges of the squares are oriented obliquely and the fundamental SF component is on the horizontal and vertical meridians (bottom). Abbreviations: Check, checkerboard pattern; Diamond, diamond pattern.

Regarding stimulus orientation, Kelly [11] reported that the stimulus orientations of the Fourier power spectra in the two-dimensional SF distribution differ according to the patterns. Similarly, De Valois and De Valois [12] reported that the square-wave gratings contained numerous higher harmonics not found in sinusoidal grating at a variety of other orientations. A two-dimensional Fourier analysis of the patterns revealed that the stimulus components of the fundamental SF consisting of 2 cycles/deg are distributed horizontally with odd harmonics in the square-wave gratings, being diagonal in Check and vertical and horizontal in the Check rotated 45° pattern (Diamond). In addition, Check and Diamond show complex spatial distributions with additional odd harmonic components of the fundamental SF (Fig 1). Later, it was shown that the edge orientation of the stimuli is not responsible for the difference between vertical gratings and Check in the diagnosis of MS [13]. Therefore, we assumed that the horizontal and vertical distribution of the SF, i.e., Diamond, has a stronger stimulating effect than the diagonal distribution of Check (orientation effect) [10,14] and more resistance to retinal blur.

In this study, we investigated the potential of VEPs to check and diamonds as an indicator of objective accommodation (OA) of power. To achieve this, we increased the retinal blur of participants by adding concave (minus) lenses to the uncyclopleged dominant eye to determine their refractive power. We defined OA as the least amount of refractive error that produced sufficient VEPs to meet a predetermined cutoff value. We hypothesized that P100 to Diamond is more resistant to induced retinal blur than that to Check because of its SF orientation, i.e., the fundamental SF is oriented vertically and horizontally (Fig 1). In addition, subjective accommodation (SA) was evaluated using a visual display terminal (VDT) near-point meter (NP-200; TOMEY, Aichi, Japan) [15]. Then, we determined the relationship between the refractive power obtained from PR-VEPs and the SA obtained using the NP-200 meter.

## Materials and methods

### Participants

**Fig 2:**
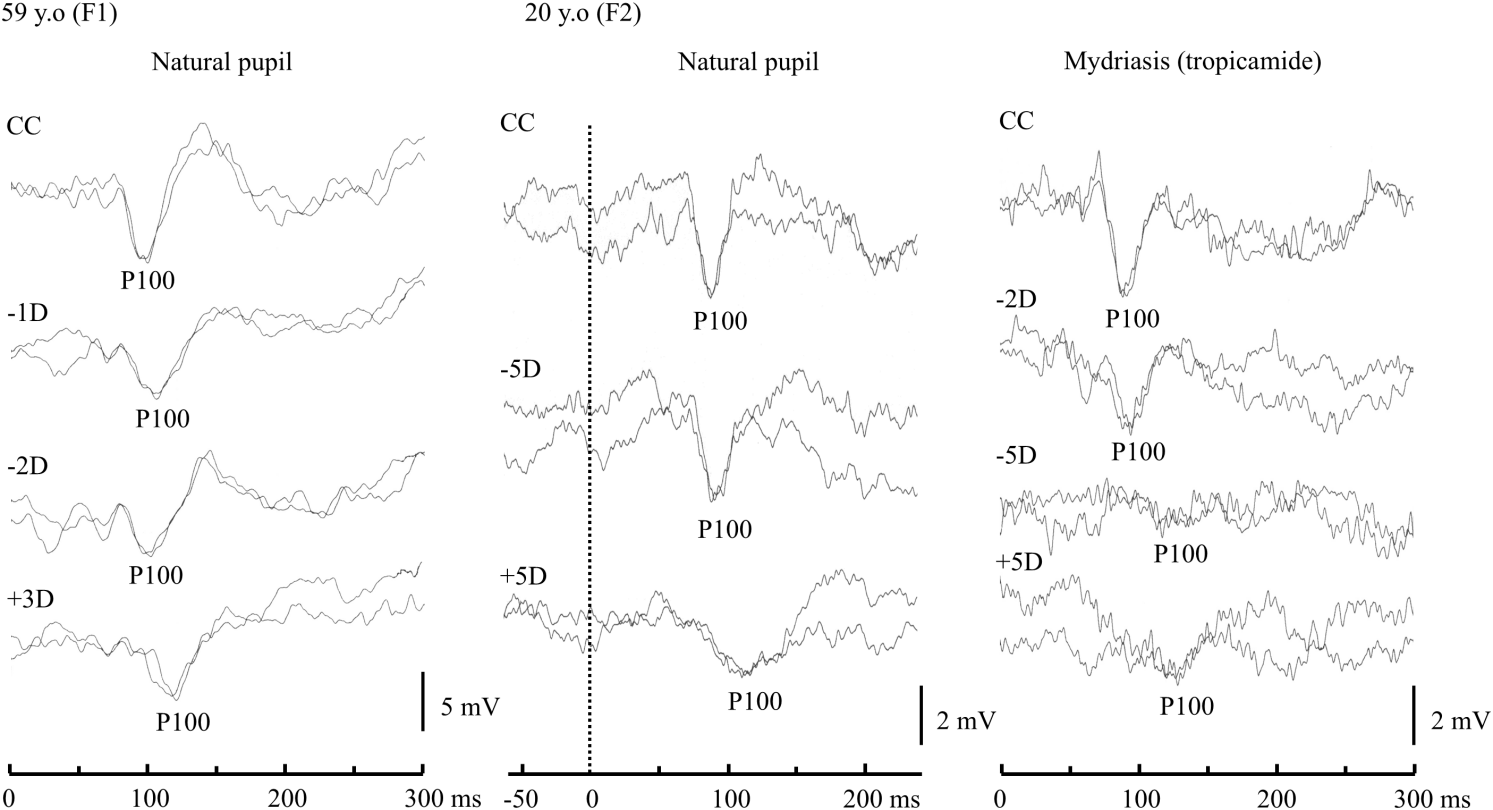
Effects of lens loading with age under natural pupil and mydriatic conditions. For both subjects, the P100 latency to Check was shortest when the VA was optimal in the CC condition (i.e., when the pattern was least blurred). Defocusing the retinal image produced alterations in VEP waveforms. Convex lens loading decreased the P100 amplitude in both female subjects examined: F1 (59 years of age) and F2 (20 years of age). Concave lens loading decreased the P100 amplitude at −2 D in F1, but did not affect the amplitude or latency at −5 D in F2. After administration of mydriatic eye drops (tropicamide) to F2, the waveforms were even affected by −2 D lens loading due to the cycloplegic effect. Please note that the difference in defocus conditions between the two participants under natural pupil conditions was due to the stronger accommodation ability of young adults. Changes in the VEP waveform were observed at −2 D in the older participant (F1), while no clear change was seen, even at −5 D, in the younger participant (F2). However, after administering a mydriatic agent to F2, changes in the waveform occurred at −2 D, similar to those observed in F1. Abbreviation: CC, cum correctione.

Before conducting the main experiment, the effects of blur caused by spherical lenses on P100 to Check were examined in two female participants (F1, 59 years of age; F2, 20 years of age) under uncyclopleged and cyclopleged eye conditions with mydriatic eye drops (tropicamide) (see Fig 2). Subsequently, 21 university students (12 women, 9 men) with corrected VA of 0.0 logMAR (logarithmic minimum angle of resolution) or better were recruited at Fukuoka International University of Health and Welfare, as participants for the main experiment. The age range of the participants was 20–22 years (20.79 ± 0.52 years, mean ± SD). The study was conducted after receiving approval from the ethics review committee of Fukuoka International University of Health and Welfare (approval number: 21-fiuhw-001). Participants were recruited from August 1, 2021 to March 31, 2023. After receiving an explanation of the study purpose and procedures, they provided informed written consent. All procedures involving human participants were conducted in accordance　with the ethical standards of the institutional and/or national research committee, as well as the 1964 Helsinki Declaration and its subsequent amendments, or other comparable ethical standards.

### Visual acuity

We used a 5m decimal visual acuity chart (VC-30; Takagi Seiko, Toyama, Japan) to obtain visual acuity. Correction was performed based on the objective refraction values detected by auto ref / keratmeter (ARK-1a; NIDEK, Aichi, Japan). Astigmatism was also completely corrected to visual acuity of at least 0.0 logMAR.

### Visual stimuli

We used Check and Diamond to compare the orientation effects of the fundamental SF on PR-VEPs. Diamond was created by rotating Check by 45° using PsychoPy^®^ (https://www.psychopy.org/index.html). This guaranteed that the spatial averaged luminance of the pattern remained unchanged when the orientation of the pattern edge was changed. The check size was 15 min of arc, in the circular visual field of 8° at a viewing distance of 114 cm. Thus, the fundamental SF of 15 min of arc was approximately 2.8 cycles/deg. All checks were square, and the contrast was 90% with a mean luminance of 50 cd/m^2^. Prior to the experiment, the luminance and contrast of the visual stimuli were calibrated using a CS-150 luminance meter (Konica Minolta, Japan). Check and Diamond were reversed at a rate of 1 Hz. Details of the LCD monitor was as follows: ASUS VG258, 24 inches, 1920 x 1080 pixels. refresh rate; 100 Hz).

### VEP recordings

**Fig 3:**
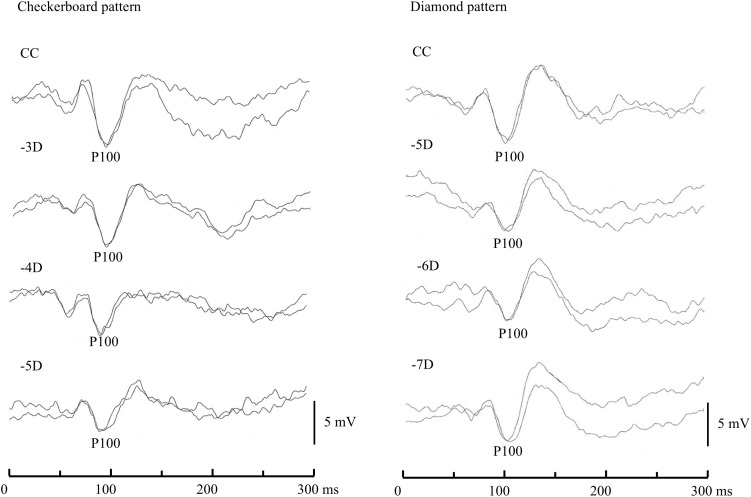
Changes in VEP waveforms after concave lens loading in a representative subject. Even when the P100 latency was unaffected, the P100 amplitude was affected by the refractive power. Note that the VEPs to Diamond were more strongly affected by the refractive power than those to Check.

PR-VEPs were recorded from the Oz-Fz derivation (international 10−10 system) in accordance with the recommendations of the International Society for Clinical Electrophysiology of Vision (ISCEV2016) [16] using a sampling frequency of 1 kHz and an analysis time of 300 ms (Neuropack; Nihon-Kohden, Tokyo, Japan). A ground electrode was placed over Cz. VEP recordings were performed using a + 0.75 D correction for near vision, in addition to the standard 5 m correction, to adjust for an observation distance of 114 cm. This condition is defined as complete correction (cum correctione: CC). Thereafter, we recorded the baseline waveform with CC spectacles from the dominant eye in the natural pupil condition (see upper traces of Figs 2 and 3). Serial VEPs were then recorded with concave lens loading. At least two trials averaging 64 responses were performed to ensure reproducibility of the PR-VEPs with each concave lens. Additionally, we were careful to maintain the participants’ attentiveness. Whenever the VEP responses became degraded, short rest breaks were taken. Then, we recorded VEPs again, to confirm reproducibility. Finally, the two trials were averaged to obtain the latency and amplitude of the P100.

### Near-point (NP) measurements

The dominant eye was determined by the hole-in-card test [17], in which eight participants showed left eye dominance and 13 participants showed right eye dominance. For SA, the NP distance was evaluated using the NP-200 [18]. This machine is manually moved to measure the point in space conjugate to the retina at which the eye is fully accommodated. NP was measured using fully corrective power lenses, whereas the far point (FP) was measured with an NP measurement power of +4 D. First, we confirmed that the fixation target appeared clear by adjusting the lens. Next, the participants gazed at the approaching fixation target while keeping it in focus so that it was not blurred. The NP of adjustment was determined as the distance from the front of the eye to the target at the limit of focus adjustment, or when the target was blurred. Meanwhile, the FP of adjustment was determined as the distance from the front of the eye to the target when the target was blurred by gazing at a distant fixation target with a convex (plus) lens added to the corrective lens. The SA was calculated by using the following equation [18]: the NP and FP values were measured five times, and the obtained average values of NP and FP were adopted.


$$\begin{array}{c} SA=\frac{\text{1}\text{0}\text{0}}{\overset{―}{NP}}-\frac{\text{1}\text{0}\text{0}}{\overset{―}{FP}}+\text{4}\left(D\right) \end{array}$$

### Data analyses

The P100 amplitude was measured from the preceding negative wave (N75). Cut-off values to calculate the refractory power were based on previous studies [19–21]. Briefly, the P100 latency was prolonged by more than 5 ms and/or decreased to less than 80% of the amplitude compared with the P100 of the baseline VEP. We defined the refractive power that met these cutoff criteria as OA. For instance, when −5 D is added under the CC condition and the P100 amplitude in the baseline VEP decreases to less than 80% of its original value, the OA is considered 5 D. Participants excluded from the analyses included those who showed waveform distortion due to noise contamination during VEP recordings and those who lacked VDT NP recordings. A linear regression analysis was performed between SA and OA. Values of *p* < 0.05 were considered to indicate statistical significance. All statistical analyses were performed using EZR software [22] and effect sizes were calculated using G*Power software [23,24].

## Results

### Effects of spherical lenses on P100 to check

Two illustrative cases are shown in Fig 2. Convex lens loading decreased the P100 amplitude in both F1 and F2 (Fig 2). Although a −2 D lens decreased the P100 amplitude in F1, a −5 D lens did not affect the P100 amplitude or latency in F2. Interestingly, even a −2 D lens affected the VEP waveforms in F2 under mydriatic conditions. The increase in retinal blur with the −5 D lens caused more severe distortion of the VEP waveforms. These results suggest that P100 is affected by convex lens loading irrespective of age and pupil conditions, whereas concave lens loading does not affect P100 in young subjects under natural pupil conditions due to their accommodation ability. The results were consistent with the findings of Otake et al. [25], who demonstrated that accommodative ability tends to decline with age.

### Main experiment

We analyzed data for 19 of the 21 participants because the data for two subjects who met the exclusion criteria were discarded.

### Comparisons of PR-VEPs to check and diamond

Representative examples of PR-VEPs to Check and Diamond are shown in Fig 3. The main VEP data in the complete correction condition are described in the following text. The P100 latencies to Check and Diamond were 97.72 ± 3.84 (mean ± SD) ms and 99.92 ± 10.08 ms, respectively, while the mean P100 amplitudes to Check and Diamond were 9.10 ± 4.87 μV and 9.67 ± 4.40 μV, respectively. The mean P100 latencies to Check and Diamond were comparable to the normal values reported by Coupland and Kirkham [26].

### OA measurements

When recorded with either stimulus, the P100 latency and amplitude were affected by refractive error, which reduced the amplitude even when the latency was unaffected by concave lens loading (Fig 3). The OA for Diamond was 5.05 ± 1.15 D, while that for Check was 4.74 ± 1.62 D.

### Comparisons of SA and OA

**Fig 4:**
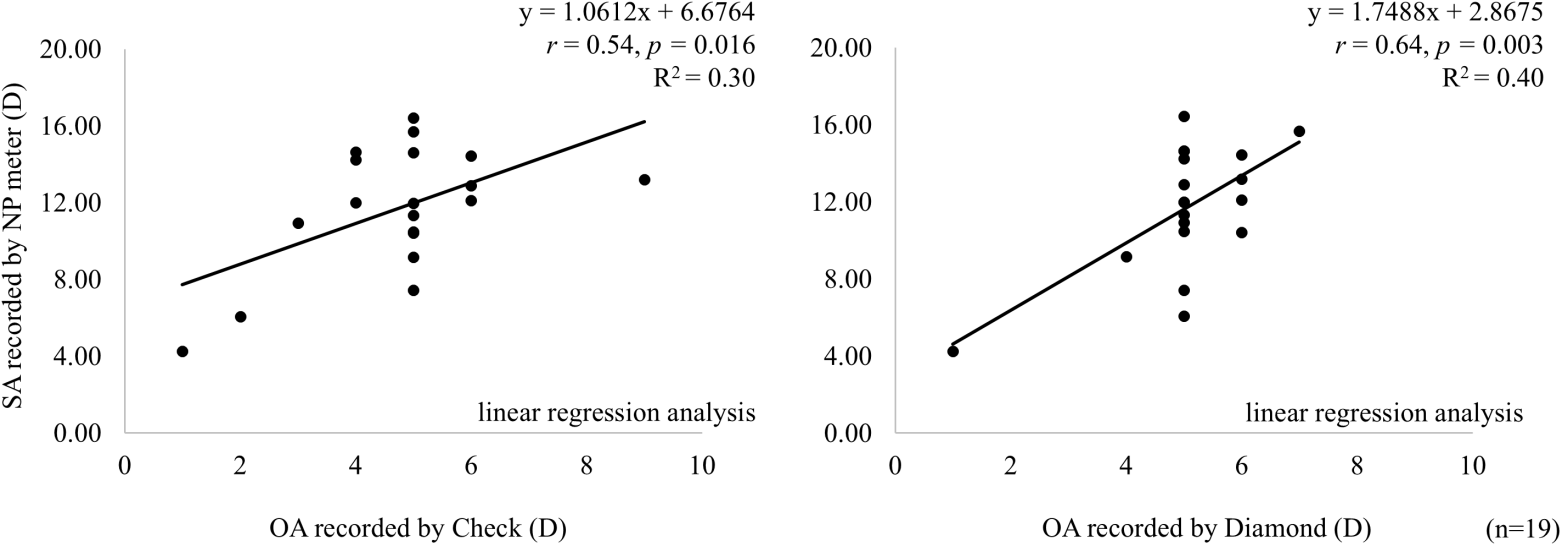
Correlations between SA obtained with the VDT NP meter and OA measured by VEPs. Significant positive linear correlations were found between OA measured by Check and SA (left), and also between OA measured by Diamond and SA (right). A post hoc power analysis showed that Diamond had sufficient statistical power, even with a sample size of 19, compared with Check.

The mean SA determined by the VDT NP meter was 11.70 ± 3.24 D. The SA values obtained using the VDT NP meter were significantly higher than the OA values obtained from VEPs to Check and Diamond. A paired t-test revealed significant differences between SA and OA values for Diamond (r = 0.64, t = −10.939, p < 0.01, effect size = 0.67, 95% confidence interval [CI] = [0.348, 2.277]) and Check (r = 0.54, t = −11.172, p = 0.01, effect size = 0.43, 95% CI = [−0.009, 1.876]). The 95% CIs were calculated using a bootstrap method with 4,000 resamples. We performed a post hoc power analysis on the simple linear regression model for Diamond and Check, respectively. For Diamond, the power was 0.92 based on degrees of freedom (df) = (1, 17) and a noncentrality parameter of 12.73. For Check, the power was approximately 0.77, based on df = (1, 17) and a noncentrality parameter of 8.17. These results indicated that Check was slightly underpowered, whereas Diamond had sufficient statistical power, even with a sample size of 19 Fig 4.

## Discussion

The main finding of this study was that VEPs to Diamond may be more resistant for retinal blur than those to Check. Although we realize that the results of only two participants cannot be generalized (Fig 2), our illustrative cases showed that the presence or absence of accommodation affected the P100 latency and amplitude. In the natural pupil condition (uncyclopleged eye), the P100 remained unchanged as long as the subject could accommodate to overcome the blur induced by concave lenses. For example, our younger adult subject (20 years of age) could overcome −5 D of blur while the older presbyopic adult (50 years of age) showed increased P100 latency and decreased P100 amplitude with a −2 D lens (Fig 2). When the eye was cyclopleged, the introduction of concave lenses had much the same effect as convex lenses, including an immediate increase in latency and decrease in amplitude. The stability of P100 under concave lens loading in the young subject is consistent with the results of previous reports [5,6]. Additionally, these results appear to result from the mechanism of presbyopia, in which accommodative ability is high in youth but decreases with age. The fact that there was a correlation between increased P100 latency or decreased P100 amplitude and clinical measurement of accommodation agrees with the work of Millodot & Newton (1981) [5] who observed that alterations in P100 caused by concave lenses may be useful to measure OA in human adults. However, these results should be interpreted with caution. They resulted from two illustrative samples of age-related changes and cannot be generalized to all populations. The report by Otake et al. [25] clearly demonstrates that accommodative ability declines with age, which may support our interpretation in these two cases.

Visual resolving power is known to be poorer for objects oriented obliquely compared with objects with horizontal and vertical orientations [10, 14]. This effect is due to some orientation inequality in the human visual system (orientation effect or oblique effect). In 1976, Kelly [11] showed that when the SF spectrum of Check with vertically (90°) and horizontally (180°) oriented edges is analyzed with Fourier techniques, the fundamental SF component does not lie on the horizontal or vertical axes but rather in the 45° and 135° (oblique) orientations (see Fig 1). Subsequently, in 1979, May et al. [9] recorded steady-state VEPs to Check and vertical gratings to examine the effects of cylindrical lenses in various orientations. When the cylinder lenses were oriented with 90° or 180° edges of Check, the VEP latency increased monotonically with increasing astigmatic blur. On the contrary, when the cylinder lenses were aligned along the 45° or 135° axis, the orthogonal was minimally affected (maximal in focus) and the latency was unchanged. When spherical lenses were used, all orientations were equally blurred, and P100 latency increased with increasing spherical power. Therefore, our findings are compatible with the hypothesis that P100 latency is dependent on the optical integrity of the fundamental SF component of Check and Diamond even though the subject is unable to consciously perceive these components when viewing a focused Check and Diamond.

In clinical practice for MS diagnosis, Coupland and Kirkham [26] reported that addition of Diamond stimulation increased the proportion of MS patients with VEP delays by approximately 11%, owing to the presence of orientation-specific abnormalities in the VEPs of some patients. These findings suggest that the difference in SF distribution between Check and Diamond is critical for assessment of VA by VEPs. Thus, we compared the influence of concave lenses on the uncyclopleged eye for VEPs to both Check and Diamond. We found that VEPs to Check may be more susceptible to retinal blur than those to Diamond. As previously mentioned, the fundamental SF of Check is oriented diagonally while that of Diamond is aligned vertically and horizontally. Retinal blur has more effect on the fundamental SF aligned along the 45° or 135° axis than on that aligned along the 90° or 180° axis [9]. Thus, the reduced contribution of the fundamental SF of VEPs to Check caused faster degradation of P100 compared with the less affected contribution of the fundamental SF of VEPs to Diamond. These findings indicate that Diamond is a more directionally selective and sensitive stimulus than Check [13,26], and that VEP can be an objective indicator of accommodation power. Nowadays, commercially available auto ref / keratometers are distributed, which can assess the accommodation power. Thus, it is necessary to determine whether the use of such devices is useful in evaluating OA in the near future.

Different views may exist regarding what constitutes an appropriate cutoff value for OA and the selection of the dominant or non-dominant eye based on the measurement. Regarding intra-individual variability, P100 amplitudes are more variable than P100 latencies. The major factors contributing to this variability are prolonged recording time and fatigue. Many previous studies have documented intra-individual variability in P100 latency, but reports on variability in P100 amplitude are scarce. In this study, the 80% amplitude criterion seemed reasonable, even though the check sizes were not exactly the same. Additionally, previous studies have reported no significant differences in P100 latency or amplitude between the dominant and non-dominant eyes [27,28]. Therefore, the effect of eye dominance on VEP measurements is considered minimal. Regarding accommodative responses, one study showed minor differences in speed between the dominant and non-dominant eyes [29,30]. However, this difference was less than 0.5 D, which is clinically negligible. Since our study used 1 D step changes, such differences are less likely to affect the results. Based on these findings, we recorded VEPs from the dominant eye in this study.

Some might argue that our results were produced by spurious resolution. Spurious resolution is a byproduct of optical defocus whereby sinusoidal image components are reversed in sign: light stripes appear dark and vice versa. Such contrast-reversal effects are readily apparent in defocused projected images, and in subjective visual experience when viewing targets closer than the NP of the eye [31]. As far as we know, previous studies have never considered the effect on VEPs of spurious resolution caused by retinal blur. In our experimental setting, the viewing distance was 114 cm, which was outside the NP of the eye. Additionally, we used Check and Diamond patterns but not sinusoidal gratings to record the VEPs. Therefore, we believe that spurious resolution is not the cause of our results.

In this study, SA was measured using an NP-200 meter. The accommodation power of SA was significantly greater than that of OA obtained from VEPs. This large difference is likely the result of the different measurement methods. First, the NP-200 meter directly measures the full range of the accommodative response; the range from the far point to the near point results in a large value. In contrast, OA estimated by VEPs is limited to the dynamic range in which electrophysiological degradation can be detected. This may cause underestimation of the actual amount of accommodation. At the same time, we cannot rule out the possibility of a physiological mechanism that compensates for retinal blur. Specifically, retinocortical gain control, a complementary function of V1 that compensates for retinal blur [32,33], may contribute to this difference. Retinocortical gain control is a mechanism by which the strength of visual signals is modulated along the pathway from the retina to the visual cortex. Additionally, it is known that cortical responses can change nonlinearly in relation to variations in visual input. Through these mechanisms, the NP-200 meter may require greater adjustments than VEPs. However, these mechanisms were not directly examined in the present study. Therefore, this remains a speculative explanation that should be interpreted with caution.

There are some limitations in this study. First, the overall experimental time was set to 1 hour, in consideration of the fatigue burden on the participants. Therefore, the VEP measurement time was limited, and we were able to record VEPs only with 1 D increments in lens power. For detailed comparison with SA, we would have preferred to measure and evaluate 0.25 D increments, based on the refractive value of the auto ref / keratometer. However, it remains possible that detailed changes in OA would not be evaluated due to the inspection time. Second, we did not measure pupil diameter using an appropriate device. Salim et al. reported that pupil diameter can reduce the signal-to-noise ratio (SNR) of VEPs [33]. However, age-related miosis is expected to be minimal in this study because all participants were young adults aged 20–22 years. Additionally, the luminance of the LCD monitor, viewing distance, and room lighting were standardized, which limits fluctuations in pupil diameter during VEP recordings. Finally, since the participants were exclusively young adults aged 20–22, further research is needed to determine whether the same results could hold true for other age groups.

## Conclusion

Our results showed that VEPs to Diamond may be more effective than those to Check for evaluating OA. This probably arose through dependence on the SF distribution of the fundamental SF component. SA produced greater adjustment than VEPs irrespective of retinal blur, which was considered to reflect the compensatory function of V1. These findings suggest that V1 may provide cortical magnification of the retinal input to adjust the retinal blur. Overall, our results provide a new insight into our understanding of vision physiology.

## Supporting information

S1 Table Raw visual evoked potential (VEP) latency and amplitude under Check and Diamond conditions, and raw NP-mater measurements for all subjects. (XLSX)
